# Superimposed Tissue Formation in Human Aortic Valve Disease: Differences between Regurgitant and Stenotic Valves

**DOI:** 10.3390/jcdd8070079

**Published:** 2021-07-08

**Authors:** Boudewijn P. T. Kruithof, Aniek L. van Wijngaarden, Babak Mousavi Gourabi, Jesper Hjortnaes, Meindert Palmen, Nina Ajmone Marsan

**Affiliations:** 1 Department of Cardiology, Leiden University Medical Center, 2333 ZA Leiden, The Netherlands; a.l.van_wijngaarden@lumc.nl (A.L.v.W.); N.Ajmone@lumc.nl (N.A.M.); 2 Department of Cell and Chemical Biology, Leiden University Medical Center, 2333 ZA Leiden, The Netherlands; 3 Department of Anatomy and Embryology, Leiden University Medical Center, 2333 ZA Leiden, The Netherlands; b.mousavi_gourabi@lumc.nl; 4 Department of Cardiothoracic Surgery, Leiden University Medical Center, 2333 ZA Leiden, The Netherlands; J.Hjortnaes@lumc.nl (J.H.); M.Palmen@lumc.nl (M.P.)

**Keywords:** aortic stenosis, aortic regurgitation, superimposed tissue, original leaflet, myofibroblasts, free edge folding

## Abstract

The formation of superimposed tissue (SIT), a layer on top of the original valve leaflet, has been described in patients with mitral regurgitation as a major contributor to valve thickening and possibly as a result of increased mechanical stresses. However, little is known whether SIT formation also occurs in aortic valve disease. We therefore performed histological analyses to assess SIT formation in aortic valve leaflets (*n* = 31) from patients with aortic stenosis (*n* = 17) or aortic regurgitation due to aortic dilatation (*n* = 14). SIT was observed in both stenotic and regurgitant aortic valves, both on the ventricular and aortic sides, but with significant differences in distribution and composition. Regurgitant aortic valves showed more SIT formation in the free edge, leading to a thicker leaflet at that level, while stenotic aortic valves showed relatively more SIT formation on the aortic side of the body part of the leaflet. SIT appeared to be a highly active area, as determined by large populations of myofibroblasts, with varied extracellular matrix composition (higher collagen content in stenotic valves). Further, the identification of the SIT revealed the presence of foldings of the free edge in the diseased aortic valves. Insights into SIT regulation may further help in understanding the pathophysiology of aortic valve disease and potentially lead to the development of new therapeutic treatments.

## 1. Introduction

Aortic valve disease is mainly divided into aortic stenosis and aortic regurgitation [1]. Stenotic aortic valves are characterized by increased leaflet stiffness and thickening due to fibrosis and calcifications, and therefore a decreased valve opening in systole with increased transvalvular pressure gradient [2]. Regurgitant aortic valves are characterized by the lack of leaflet coaptation during diastole, secondary to aortic root or ascendens dilatation or by primary leaflet abnormalities such as myxoid degeneration or post-inflammatory fibrosis [1]. These aortic valve diseases have a very different pathophysiology, but in both cases alterations of valve stresses are believed to play an important role in the initiation of leaflet abnormalities, as well as in the propagation of leaflet remodeling [3].

The aortic valve leaflet consists of 3 layers: the fibrosa, spongiosa, and ventricularis, which are rich in collagen, proteo- and glycosaminoglycans, and elastin, respectively [4]. These layers are demarcated by the elastin layer of the ventricularis on the ventricular side and by a thin elastic lamina on the aortic side of the valve. Valvular endothelial cells and their basement membrane surround these elastic lamina, whereas valvular interstitial cells are interspersed within the aortic valve. The hallmark of a stenotic aortic valve is the presence of calcification, which is thought to start in the fibrosa layer but might expand toward the other leaflet layers [2]. Leaflets of regurgitant aortic valves in turn typically display degeneration of the extracellular matrix, leading to a weakened leaflet [1]. Recently, the formation of superimposed tissue (SIT), an extra layer of tissue on top of the original leaflet, has been described as a major component of valve thickening and remodeling in patients with mitral valve prolapse [5,6]. Using an ex vivo animal model, SIT was suggested to be the result of increased mechanical and hemodynamic stresses [5]. SIT-like lesions, with tissue expansion outside the elastic lamina, also have been described in autoptic studies assessing early stages of aortic stenosis [7,8], but have not been systematically characterized or, more importantly, examined in advanced aortic valve disease.

We therefore hypothesized that SIT formation might frequently occur in aortic valve disease and most probably in both aortic regurgitation and stenosis, being both characterized by changes in mechanical and hemodynamic stresses. By examination of aortic valves resected from patients with significant aortic regurgitation or stenosis, we systematically observed SIT formation on both the aortic and ventricular sides of the leaflet and in both regurgitant and stenotic aortic valves. However, the distribution and composition of the SIT significantly differed between the regurgitant and stenotic valves, likely due to the difference in mechanical stresses between the two pathologies.

## 2. Materials and Methods

### 2.1. Human Aortic Valve Samples

Aortic valve samples were collected from patients (*n* = 20) who underwent aortic valve replacement because of moderate/severe stenosis (*n* = 12) or moderate/severe regurgitation due to aortic dilatation (*n* = 8). We selected regurgitant valves caused by aortic dilatation in order to determine specifically the effect of changed hemodynamic flow patterns on the SIT formation. From each patient, one or more aortic valve leaflets were included for analysis, resulting in a total of 17 leaflets from stenotic valves and 14 leaflets from regurgitant valves. The samples were fixed overnight in a 4% paraformaldehyde solution in phosphate buffered saline (PFA/PBS, pH 7.2). Collection and analysis of the samples were performed according to the guidelines of the Leiden University Medical Centre (Leiden, The Netherlands) and according to the Dutch regulations on the use of human tissues (rest material). Furthermore, the study was conducted according to the principles of the Declaration of Helsinki (64th WMA General Assembly, Fortaleza, Brazil, October 2013) and national and institutional guidelines, regulations, and acts.

### 2.2. Histological Analysis

Human valve tissue was processed as previously described [5]. Briefly, the valve samples were dehydrated through a graded series of ethanol, cleared in xylene, embedded in paraffin, and sectioned at 6 μm, producing longitudinal sections oriented from the leaflet base to the free edge. Sections were stained according to the manufacturer’s protocol with Weigert’s Resorcin Fuchsin (EMS, Hatfield, PA, USA) to identify elastic fibers, and counterstained with nuclear-fast red (Sigma, Zwijndrecht, The Netherlands). Sections were stained using Masson’s Trichrome (Masson’s trichrome kit, Klinipath, Duiven, the Netherlands) to visualize collagen fibers, and Alcian blue (Klinipath) to visualize glycosaminoglycans. For immunofluorescent staining, the slices were deparaffinized, hydrated, and boiled 35 min in antigen retrieval buffer (10 mM Tris (pH9)/1mM EDTA/0.05% Tween-20) using a pressure cooker. After blocking with 1% BSA in 0.1% Tween-PBS, sections were incubated overnight with primary antibodies against alpha-smooth muscle actin (αSMA; 1:10,000, A2547, Sigma), platelet endothelial cell adhesion molecule (PECAM-1; 1:1000, AF3628, R&D, Abingdon, UK), laminin (1:200; Z0097, Dako, Santa Clara, CA, USA), collagen IV (1:100; 1340-01, SouthernBiotech, Birmingham, AL, USA), or Ki67 (1:100, AB9260, Millipore, Amsterdam, the Netherlands), followed by incubation with an alexa-conjugated secondary antibody (Thermo Fisher, Bleiswijk, the Netherlands). Slides were mounted using DAPI containing Prolong Gold Antifade reagent (Thermo Fisher). All slides were scanned with the Pannoramic 250 slide scanner (version 1.23, 3DHISTECH Ltd., Budapest, Hungary) and analyzed using Caseviewer (version 2.4, 3DHISTECH Ltd.).

### 2.3. Measurements

**Figure 1:**
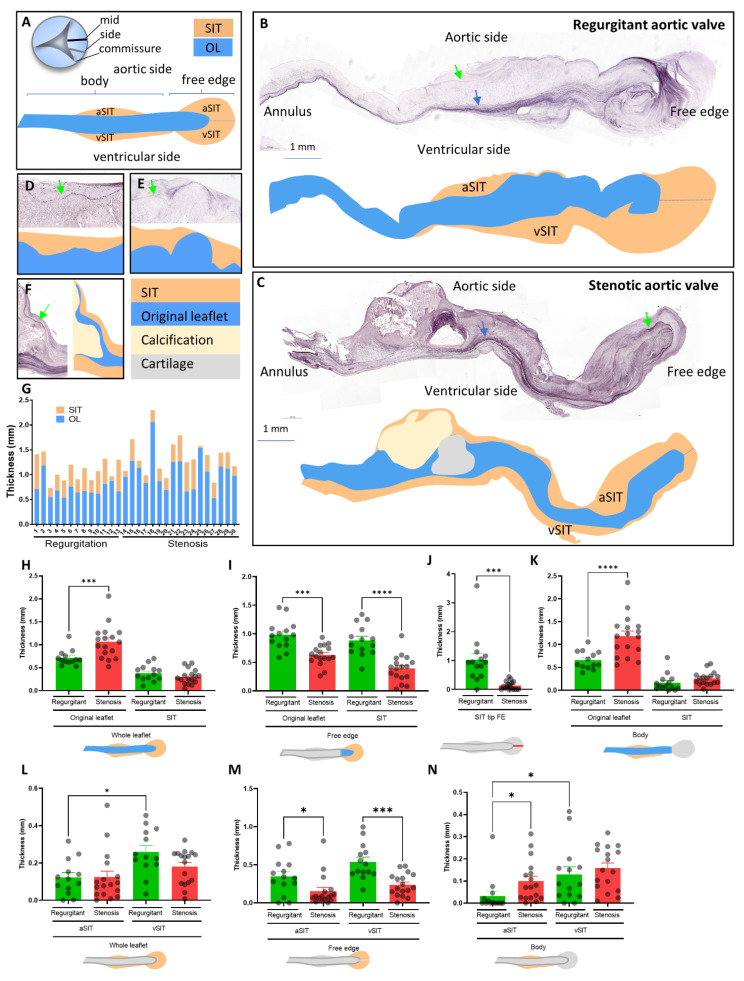

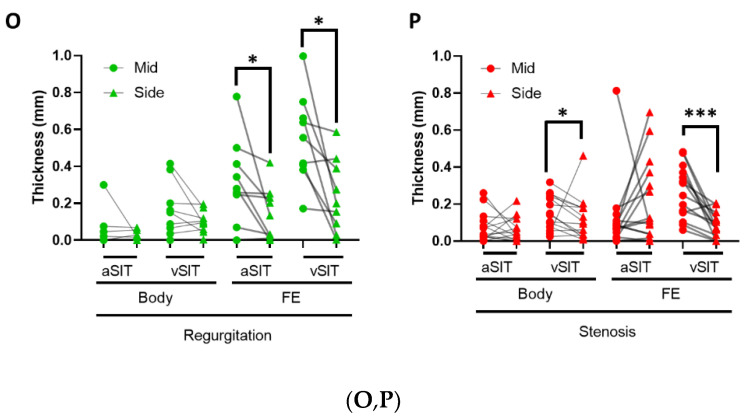
SIT distribution in regurgitant and stenotic valves. (**A**) schematic drawings indicating the defined regions of the aortic valve. (**B**–**F**) Elastin stainings indicating the borders between the original leaflet (OL) and the SIT on the ventricular (blue arrow) and aortic side (green arrow) of the aortic leaflet, and rendering of the same leaflet with blue representing the original leaflet, orange representing the SIT, yellow representing the calcific region, and gray representing the cartilaginous region. (**G**) Graph indicating the contributions of the SIT and OL to the average thickness of each leaflet. (**H**–**N**) Graphs indicating the average thicknesses of the indicated regions in the regurgitant and stenotic leaflets. (**O**,**P**) Graphs depicting the comparisons of the average thicknesses of the SIT at the midline of the aortic valve (mid) with the region closer to commissure (side) at the indicated parts of the regurgitant (**O**) and stenotic valve (**P**). Data are presented as means ± SEM. One-way ANOVA followed by Šidák’s multiple comparisons test was performed for graphs (**H**,**I**,**K**–**M**), an unpaired *t*-test was performed for graph (**J**), and a Kruskal-Wallis test followed by Dunn’s multiple comparisons test was performed for graph N. The Wilcoxon matched-pairs signed rank test was performed for graphs (**O**,**P**) to evaluate significant differences. * *p* < 0.05, *** *p* < 0.001, **** *p* < 0.0001. Scale bar is 1000 µm.

Measurements of original leaflet and SIT thickness were performed on sections stained for elastin at the midline or in between the midline and the commissure of the aortic valve leaflet (see indications in Figure 1A) using Caseviewer software. The surfaces of the original leaflet, of the SIT on the ventricular side of the aortic valve (ventricular SIT, vSIT) and of the SIT on the aortic side of the aortic valve (aortic SIT, aSIT), were measured at the level of the leaflet body and of the free edge, and divided by the length of the leaflet (either whole leaflet or original leaflet) to obtain the respective average thickness (Figure 1A). ImageJ software was used to determine the intensity of Masson’s trichrome and Alcian blue staining and the αSMA-positive area. For each parameter, only the valve samples that allowed measurement of the complete indicated area(s) were included.

### 2.4. Statistical Analysis

GraphPad Prism 9 was used for statistical analysis. Data were tested for significance as indicated in each legend using analysis of variance (ANOVA) with Šidák’s correction or Kruskal-Wallis with Dunn’s correction for multiple groups and unpaired *t*-test or the Wilcoxon matched-pairs signed rank test for comparison of 2 groups, and correlations were determined using the Pearson’s r-correlation test. Data are reported as means ± SEM. A *p*-value below 0.05 was considered significant.

## 3. Results

### 3.1. SIT Distribution in Regurgitant and Stenotic Aortic Valves

To determine whether SIT contributes to the thickness of the aortic leaflet, sections from the midline of the aortic leaflet (Figure 1A) were stained for elastin to allow the visualization of the borders of the original leaflet. A thick elastin layer outlined the ventricularis of the valve (blue arrow in Figure 1B,C), which defined the border of the original leaflet at the ventricular side. The border of the original leaflet at the aortic side of the valve was in turn defined by a relatively thin elastin layer (green arrow in Figure 1B–F). In both regurgitant and stenotic aortic valves, SIT was observed on the ventricular side (vSIT) and on the aortic side (aSIT; Figure 1A–C) of the leaflet and was found to contribute up to 50% of the total average thickness of the aortic leaflet (Figure 1G). SIT distribution was variable, from rather focal (Figure 1B) to diffuse throughout the entire leaflet, and from proximal (near attachment to the annulus) to distal (free edge; Figure 1C). Additionally, SIT appeared to smoothen the irregular surface of the original leaflet (Figure 1D,E) and in stenotic valves was found to cover large parts of the calcified regions (Figure 1C,F).

Although the average thickness of the original leaflet was significantly larger in stenotic valves (Figure 1H), the average SIT thickness did not differ between regurgitant and stenotic valves (Figure 1H). However, when dividing the leaflet in a body and free edge part (Figure 1A), regurgitant valves showed a significantly thicker free edge compared to stenotic valves due partly to a thicker original leaflet, but mostly to a thicker SIT (Figure 1I). In particular, the SIT in the tip of the free edge (stippled line in Figure 1A–C) was more than 7 times thicker in regurgitant valves compared to stenotic valves (average 1.07 mm, SEM: ±0.23 for regurgitant valves, average 0.14 mm, SEM: ±0.03 for stenotic valves; Figure 1J). In turn, stenotic valves showed a thicker body part due to a thicker original leaflet but not to a thicker SIT (Figure 1K). Although the overall thickness of the aSIT and the vSIT did not differ between regurgitant and stenotic valves (Figure 1L), the vSIT and aSIT at the level of the free edge was thicker in regurgitant valves (Figure 1M), whereas the aSIT was thicker in stenotic valves at the level of the leaflet body (Figure 1N).

The extent and types of mechanical stresses that the aortic valve experiences might be different in the regions more distant from the midline of the aortic valve [3,9,10]. To determine whether this has consequences for the extent of SIT formation, the thicknesses of the SIT at the midline of the valve (mid) were compared to the thicknesses closer to the commissure of the valve (side; Figure 1A) within each regurgitant and stenotic valve. The aSIT and vSIT at the free edges of regurgitant valves were found to be thinner closer to the commissure (Figure 1O). The vSIT of the body part and free edge part of stenotic valves was also thinner in the regions closer to the commissure (Figure 1P). Interestingly, the aSIT of the free edge appeared to be thicker closer to the commissure in the stenotic valves (Figure 1P).

The contribution of SIT to the thickness of aortic valves therefore greatly varies depending on the pathology and the specific location on the leaflet (aortic vs. ventricular; body vs free edge; midline vs. side).

### 3.2. Extracellular Matrix Composition of the SIT in Regurgitant and Stenotic Aortic Valves

**Figure 2:**
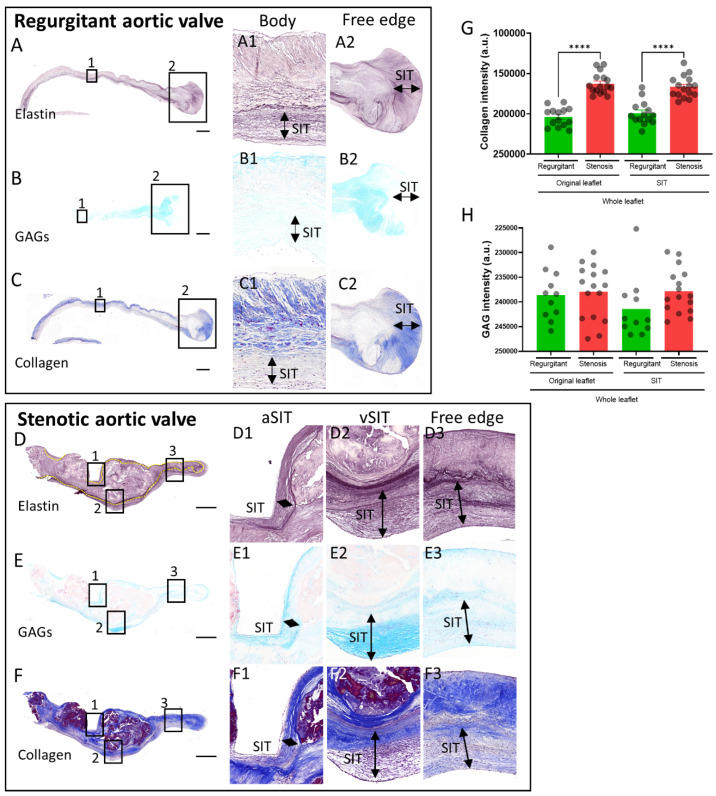
Extracellular matrix composition of SIT in regurgitant and stenotic valves. Stainings for elastin (**A**,**D**), glycosaminoglycans (GAGs; *(***B**,**E**), and collagen (**C**,**F**) on regurgitant (**A***–***C**) and stenotic aortic leaflets (**D***–***F**). *(***G**,**H**) Graphs of the collagen (**G**) and GAGs (**H**) staining intensities in the original leaflet and SIT of regurgitant and stenotic aortic leaflets. Data are presented as means ± SEM. One-way ANOVA followed by Šidák’s multiple comparisons test was performed to evaluate significant differences for graphs G and H. **** *p* < 0.0001. Scale bar is 1000 µm.

To determine the extracellular matrix composition of SIT, stainings for elastin fibers, collagen fibers (Masson’s trichrome), and glycosaminoglycans (Alcian blue) were performed (Figure 2). SIT was found to have a wide range of extracellular matrix component expression, with regions characterized by high levels of glycosaminoglycans (Figure 2(E1,E2)) or collagens (Figure 2(C2,F1,F2)) and thick layers of fragmented elastin (Figure 2(A2,D3)), and other regions in turn characterized by relatively low levels of glycosaminoglycans (Figure 2(B1,B2,E3)) or collagens (Figure 2(C1,F2,F3)) and lower amounts of elastin (Figure 2(A1)). However, on average, the collagen content in SIT was comparable to the collagen content in the original leaflet (Figure 2G), but was higher in stenotic valves as compared to regurgitant valves (Figure 2G). In turn, no significant difference in glycosaminoglycan intensity was found between stenotic and regurgitant valves (Figure 2H).

### 3.3. Presence and Distribution of Myofibroblasts in Regurgitant and Stenotic Aortic Valves

**Figure 3:**
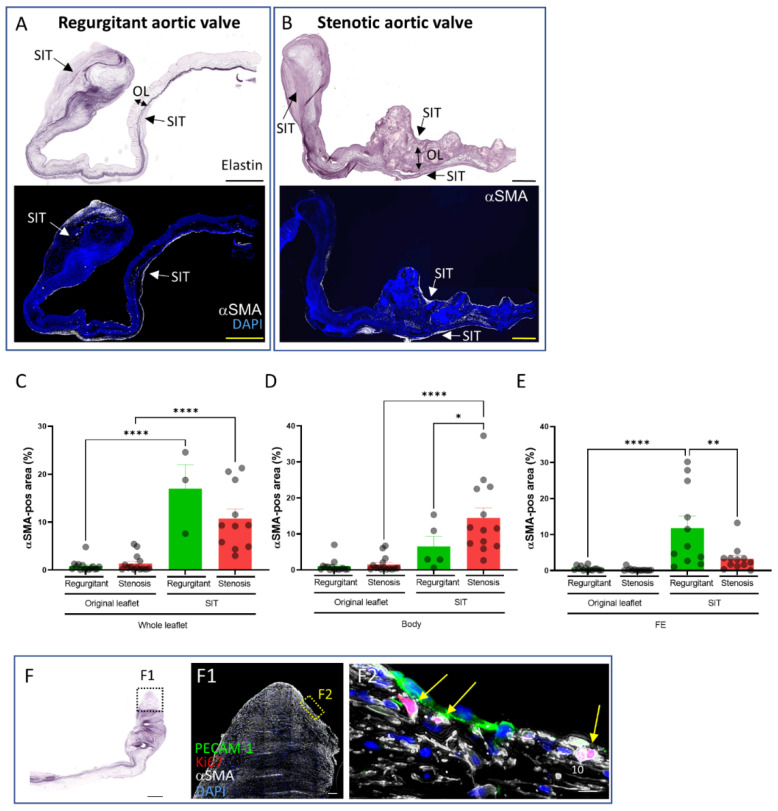
Presence and distribution of myofibroblasts in regurgitant and stenotic aortic valves. *(***A**,**B**) Elastin and αSMA stainings on regurgitant (**A**) and stenotic (**B**) aortic valves. *(***C***–***E**) Graphs indicating the αSMA-positive area of the indicated regions in the regurgitant and stenotic leaflets. (**F**) Elastin staining of a regurgitant valve (**F**) and PECAM-1, Ki67, and αSMA-co-staining (**F1**,**F2**) of the area indicated by box in F. Yellow arrows indicate Ki67-positive myofibroblasts. Data are presented as means ± SEM. One-way ANOVA followed by Šidák’s multiple comparisons test was performed to evaluate significant differences for graphs *(***C***–***E***)*. * *p* < 0.05, ** *p* < 0.01, **** *p* < 0.0001. Scale bar is 1000 µm.

Diseased aortic valves are known to be characterized by an increased presence of activated valvular interstitial cells [11,12], i.e., myofibroblasts characterized by the expression of αSMA. To determine their distribution within the SIT and the original leaflet in regurgitant and stenotic valves, αSMA staining was performed and the αSMA-positive area was measured. Myofibroblasts were predominantly observed in the SIT as compared to the original leaflet in both regurgitant and stenotic aortic valves (Figure 3A–C). However, the distribution of myofibroblasts within the SIT differed between regurgitant and stenotic valves. Whereas the SIT of the body part of stenotic valves had a larger αSMA-positive area as compared to regurgitant valves (Figure 3D), the SIT at the free edge of regurgitant valves had a larger αSMA-positive area as compared to stenotic valves (Figure 3E). To determine whether the myofibroblasts in SIT proliferate [13,14,15], Ki67 staining was performed (Figure 3F). Single Ki67-positive myofibroblasts were observed throughout the SIT, whereas groups of Ki67-positive myofibroblasts were observed in the free edge of regurgitant valves (3 out of 4), indicating an ongoing active expansion of the SIT (yellow arrows in Figure 3(F2)).

### 3.4. Comparison of the Subendothelial Basement Membrane in Regions of Regurgitant and Stenotic Aortic Valves with and without SIT

**Figure 4:**
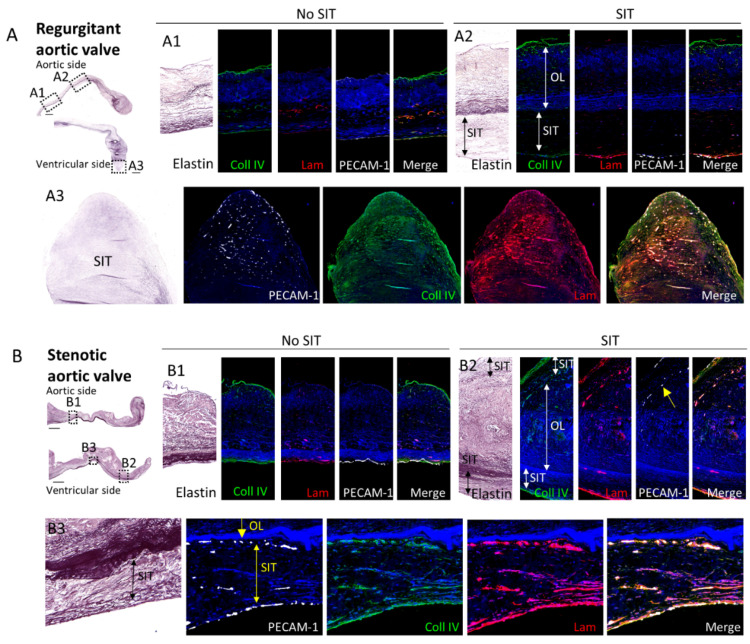
Comparison of the subendothelial basement membrane in regions of regurgitant and stenotic valves with and without SIT. Regurgitant (**A**) and stenotic (**B**) aortic valves stained for elastin, collagen IV (Coll IV), laminin (Lam), and PECAM-1 in regions without SIT (**A1**,**B1**) and with SIT (**A2**,**B2**). A3 and B3 indicate regions with SIT in the free edge of the regurgitant valve (**A3**) and in the ventricular SIT of the body part of the stenotic valve (**B3**), where the expression of collagen IV, laminin, and PECAM-1 is not confined to the luminal side but is present within the SIT. Yellow arrows in B2 and B3 indicate PECAM-positive cells at the border of the original leaflet and SIT. Scale bar is 1000 µm.

To determine whether the SIT was located beneath or on top of the endothelial lining, stainings for endothelial cell marker PECAM-1 and for subendothelial basement membrane markers collagen IV and laminin [10] were performed (Figure 4). Differences in the expression of these markers were found in regions of regurgitant and stenotic valves with or without SIT. In the regions without SIT, collagen IV was found in both aortic and ventricular basement membranes, with lower expression on the ventricular side in regurgitant valves (*n* = 4; Figure 4(A1)), but similar expression levels in stenotic valves (*n* = 5; Figure 4(B1)), while laminin was predominantly expressed on the ventricular side in both regurgitant and stenotic valves and hardly present on the aortic side (*n* = 4 for regurgitant valves; *n* = 5 for stenotic valves; Figure 4(A1,B1). In the regions with SIT, the expression of PECAM-1, collagen IV, and laminin was mostly observed on the luminal side of the SIT (either ventricular or aortic), suggesting that SIT forms beneath the endothelial lining (Figure 4(A2,B2)). The laminin expression was in most parts higher than at the regions without SIT on both the aortic and ventricular sides (*n* = 4 for regurgitant valves; *n* = 6 for stenotic valves; Figure 4(A1,A2,B1,B2)). PECAM-1, collagen IV, and laminin expression were, however, also observed dispersed within the SIT mostly in the free edge of regurgitant valves (Figure 4(A3)) andat the border of the original leaflet and the SIT in the body part of the stenotic valves (Figure 4(B2,B3)).

### 3.5. Characterization of Original Leaflet Folding in the Free Edge of Regurgitant and Stenotic Aortic Valves

**Figure 5:**
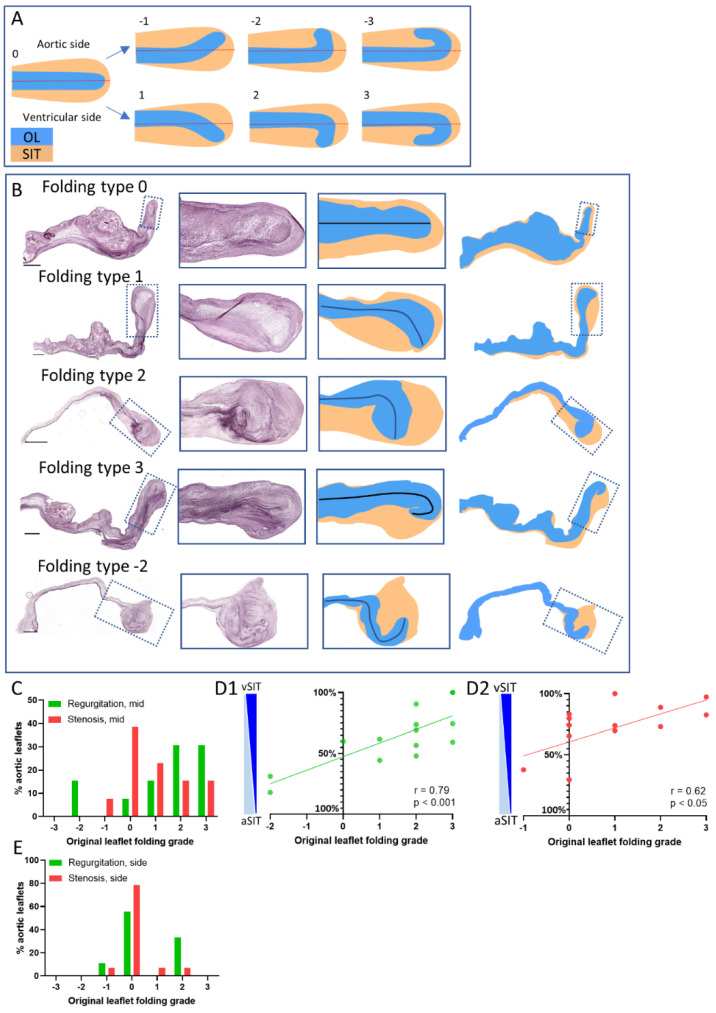
Characterization of the original leaflet folding in the free edge of regurgitant and stenotic valves. (**A**) Schematic drawings depicting the original leaflet folding types. The grade is based on the direction and the extent that the original leaflet deviates from the course of the free edge part of the leaflet, which is indicated by the red line. A grade 0 indicates no folding, whereas values above 0 indicate folding toward the ventricular side and values below 0 indicate folding toward the aortic side. The red line also indicates the border between the ventricular SIT (vSIT) and aortic SIT (aSIT). (**B**) Elastin stainings of aortic valves displaying the different folding types and the rendering of these leaflets, with blue representing the original leaflet and orange representing the SIT. (**C**) Graph showing the relative distribution of the original leaflet folding grades at the midline of regurgitant and stenotic aortic valves. (**D**) Graphs depicting the relative contribution of the vSIT and aSIT to the total SIT of the free edge in regurgitant (**D1**) and stenotic (**D2**) valves per original leaflet folding grade. (**E**) Graph showing the relative distribution of the original leaflet folding grades on the side region of regurgitant and stenotic aortic valves. Correlations between the relative contribution of the vSIT and aSIT to the total SIT and the original leaflet folding grade was determined using the Pearson’s r-correlation test. Scale bar is 1000 µm.

Examination of the free edge morphology on elastin-stained sections showed that the original leaflet part of the free edge can be folded toward the ventricular side or toward the aortic side (Figure 5). However, this folding was mostly masked by the presence of SIT surrounding the original leaflet (Figure 5). We therefore defined 7 potential grades of free edge folding (Figure 5A) to describe this phenomenon and compare it between stenotic and regurgitant valves (Figure 5B). Folding of the original leaflet in the free edge was observed in most of the regurgitant leaflets (93%; all original leaflet folding grades except grade 0) and the direction in 83% of the cases was toward the ventricular side with different grades (folding scores 1,2, and 3; Figure 5B,C). In 17% of the cases the tip of the original leaflet was folded to the aortic side (folding scores −1, −2, and −3; Figure 5B,C). On the other hand, stenotic valves did not show folding of the original leaflet in the free edge in 38% of the cases (folding grade 0), and only 1 sample showed a slight folding to the aortic side (Figure 5B,C).

In addition, the type and grade of folding of the original leaflet was correlated with the relative contribution of the SIT to the ventricular or aortic side of the free edge. The vSIT and aSIT in each folding type was defined as indicated by a red stippled line in Figure 5A along the line of the leaflet. A strong correlation was found for the regurgitant valves, with relative larger vSIT when the original leaflet was folded toward the ventricular side (Figure 5(D1)), and larger aSIT when the original leaflet was folded toward the aortic side (Figure 5(D1)). For the stenotic valves in turn, only a weak correlation was found (Figure 5(D2)).

The folding was less and often absent in the regions closer to the commissure (Figure 1A and Figure 5E) compared to the midline location in both the regurgitant and stenotic aortic valves (Figure 5C), suggesting that hemodynamic stresses are likely involved in the regulation of original leaflet folding in the free edge.

## 4. Discussions

In this study, we reported for the first time the systematic presence of SIT in both regurgitant and stenotic aortic valves. Differences in the extent, location, and composition of SIT were found between stenotic and regurgitant aortic valves, suggesting the involvement of mechanical and hemodynamic stresses in the initiation and progression of SIT formation.

### 4.1. Aortic Valve SIT Formation

The healthy aortic valve is demarcated by the aortic and ventricular elastin lamina, which are only separated from the aortic and ventricular lumen by a layer of valvular endothelial cells and their basement membrane. In cases of aortic valve pathology, most leaflet alterations (such as calcification or myxoid degeneration) occur within the elastic lamina, leading to an expansion of leaflet layers and valve thickening. However, observations of tissue expansion outside the elastic lamina also were made by Otto et al. [8] in sclerotic and stenotic aortic valves on the aortic side, being considered subendothelial lesions with a displacement and/or reduplication of the elastic lamina. Similar observations were made by Kuusisto et al. [7], who observed atherosclerosis-like lesions on the aortic side of the aortic valve of non-stenotic young and elderly patients after necropsy. Interestingly, the subendothelial lesions that were observed on the aortic surface of non-stenotic aortic valves were characterized by inflammatory cell infiltration, lipid deposition, and micro-calcifications, indicating these regions as potential initiation points of the disease. The presence of tissue outside the elastin lamina has recently also been observed on the ventricular side of early stenotic aortic valves [11]. Therefore, the presence of an additional tissue superimposed on the original leaflet, i.e., SIT, has been already suggested, especially in the early phase of aortic stenosis, but has not been systematically assessed and characterized in patients with advanced stenotic valves or in aortic valve regurgitation.

In this study, which included a large sample of aortic valves of patients with moderate or severe stenosis or regurgitation (secondary to aortic dilatation), we observed in every sample the presence of SIT formation on either the aortic, the ventricular, or both sides of the leaflet. Although no significant difference was found in the average SIT thickness between regurgitant and stenotic valves, the distribution of the SIT differed between the two pathologies. Regurgitant aortic valves showed more SIT formation in the free edge, leading to a thicker leaflet at that level, while stenotic aortic valves had relatively more SIT formation on the aortic side of the body part of the leaflet. These findings might be explained by the different mechanical stresses that stenotic and regurgitant valves are subjected to, and which are responsible for SIT formation [5]. The regurgitation etiology of the aortic valves used in the current study was aortic dilatation (and not a primary defect of the aortic valve). Leaflet remodeling of these regurgitant aortic valves, including the formation of SIT, was therefore likely caused by the hemodynamic stresses related to the dilatation and (central) regurgitation. The observed relatively high levels of SIT formation on the leaflet free edge, which was mostly subjected to the regurgitant flow, supports this hypothesis. The examined stenotic aortic valves had in turn a primary leaflet alteration (calcifications and fibrosis) causing pathologic remodeling of specifically the body part of the valve. This remodeling has been shown to change the mechanical properties of the valve and therefore the hemodynamic and mechanical stresses that the valve experiences [3]. SIT formation at this level might therefore also be initiated by hemodynamic and mechanical stress, which could explain the differential distribution of SIT between stenotic and regurgitant aortic valves. The presence of overall less SIT in the valvular regions closer to commissures also indicates the involvement of hemodynamics in SIT formation, as these regions are exposed to lower hemodynamic stresses [16,17]. Only the aSIT of the stenotic valves at the free edge part showed more SIT formation, which might suggest exposure to a different type of mechanical stress compared to the regurgitant valves.

### 4.2. Aortic Valve SIT Composition

The extracellular matrix composition of SIT greatly differed between and within the aortic leaflets, which was likely due to the “age” of the SIT and to the different mechanical and molecular stimuli each region was exposed to, as suggested by for the SIT formation in the mitral valve [5]. Some regions were shown to be highly cellular and consisted mostly of glycosaminoglycans, whereas other regions had a lower cell density and consisted mostly of collagen. Of interest, we observed a relatively higher expression of collagen in the SIT of the stenotic valves as compared to regurgitant ones, which might indicate that fibrosis in the SIT contributes to overall leaflet fibrosis and therefore to the mechanical properties of stenotic aortic valves.

Myofibroblasts are known to be present in diseased aortic valves and are thought to be the main cell type that actively remodels the extracellular matrix by the degradation and secretion of extracellular matrix components [12]. Both in stenotic and regurgitant valves, we observed that myofibroblasts, as identified by αSMA expression, are highly present in SIT but not in the original leaflet, indicating that the SIT is the more active area of the leaflet in terms of extracellular matrix remodeling in end-stage diseased aortic valves. Proliferation of these myofibroblasts in some regions of the valve at the time of isolation suggests that expansion of the SIT is still occurring in end-stage diseased aortic valves. Interestingly, αSMA cells were hardly observed in the SIT-like subendothelial lesions in stenotic aortic valves in the early disease stage [7,8].

### 4.3. Mechanisms of Aortic SIT Formation

To obtain more insights into the mechanisms of SIT formation in aortic valves, stainings for the basement membrane markers collagen IV and laminin were performed. In most regions, SIT was present underneath the basement membrane, which had increased levels of laminin compared to the regions without SIT. However, we also observed regions where valvular endothelial cells and the basement membrane markers were present at the border between the original leaflet and the SIT or dispersed throughout the part of the SIT area with αSMA expression (presumably with active remodeling). These observations in the aortic valve support the hypothesis formulated on the basis of the mitral valve ex vivo mouse model [5], that migration of activated valvular interstitial cells (αSMA-positive) and resident macrophages through breakages in the endothelial lining are responsible for SIT formation on top of the endothelial cell layer. The fact that most regions with SIT were also covered by valvular endothelial cells might indicate that these cells migrate (possibly from the sides) on top of the newly formed SIT, whereas the valvular endothelial cells within or underneath the SIT disappear over time. Future in vivo and ex vivo studies should focus on SIT formation at different time points to gain more insight into the precise mechanisms.

### 4.4. Aortic Valve Free Edge Folding

While examining the free edges of the diseased aortic valves, we frequently observed folding of the original leaflet of various degrees toward either the ventricular or aortic side. This characteristic has never been reported before and was not visible by gross examination due to the formation of SIT around the folding. The folding occurred more frequently in the regurgitant valves and was mostly directed toward the ventricular side, suggesting that the regurgitant flow caused the folding of the original leaflet. In the regions closer to the commissure, the folding was reduced or absent, supporting the involvement of the flow in the formation of the folding.

### 4.5. Limitations

Our study suggests that SIT formation is driven by hemodynamic and mechanical stresses; therefore, differences in SIT distribution and composition were expected between the aortic valve samples from patients with moderate or severe regurgitation or stenosis. The number of samples with moderate stenosis or regurgitation in our study was, however, too small to perform a robust comparison, which will therefore be included in future studies. In addition, to further characterize the composition of the SIT and substantiate differences between SIT formation in regurgitant and stenotic aortic valves, additional stainings for different extracellular matrix components are needed. In our study, αSMA staining was used to identify myofibroblasts; smooth muscle cells, however, also express αSMA and have been shown to be present in normal aortic valves and more abundant in calcified aortic valves [18,19]. Expression studies using smooth muscle cell-specific markers should be performed to distinguish the two cell types.

## 5. Conclusions

Overall, we have shown that SIT is a major feature of stenotic and regurgitant valves, contributing greatly to the thickness of the leaflets. The SIT appeared to be a highly active area with various extracellular matrix compositions potentially modifying the mechanical properties and therefore the function of the valves. The distribution and composition of the SIT differed between stenotic and regurgitant valves, indicating disease-specific regulation of SIT formation. Understanding the mechanisms underlying SIT formation is crucial to understand the initiation and progression of aortic valve disease and might open new pathways to the development of new therapeutic treatments.

## Data Availability

No report of any data.
